# Prospective analysis of grip strength and load distribution after surgical treatment of common diseases of the hand with novel’s manugraphy^®^ system

**DOI:** 10.1007/s00402-023-04984-x

**Published:** 2023-07-24

**Authors:** Jasmin S. Grüner, Aijia Cai, Isabel Pingel, Raymund E. Horch, Justus P. Beier, Andreas Arkudas

**Affiliations:** 1 Department of Plastic and Hand Surgery, Friedrich Alexander University Erlangen-Nuremberg FAU, Krankenhausstr 12, 91054 Erlangen, Germany; 2 Department of Plastic Surgery and Hand Surgery - Burn Center, University Hospital RWTH, Aachen, Germany

**Keywords:** Cylinder grip, Manugraphy^®^ system, Grip strength, Carpal tunnel syndrome, A1 annular pulley stenosis, Dupuytren’s contracture

## Abstract

**Background:**

Carpal tunnel syndrome, A1 annular pulley stenosis and Dupuytren’s contracture are among the most common conditions of the hand. In this study, we investigated the impact of surgical procedure on hand grip strength and high-resolution spatial load distribution in individuals suffering from those diseases over a follow-up period of one year.

**Materials and methods:**

In this prospective study, data of 9 patients with carpal tunnel syndrome, 12 patients with A1 annular pulley stenosis and 7 patients with Dupuytren’s contracture were evaluated. Only patients with unilateral disease were included providing the contralateral hand as an intra-individual control. Grip strength was measured with cylindrical instruments in two different sizes with respect to the hand size of the patients. Maximum and average values of grip strength as well as spatial load distribution in each finger, thenar, hypothenar and palm were analyzed. Data of the affected patients were collected preoperatively and 6 weeks, 6 months and 1 year postoperatively. Grip strength and spatial load distribution were compared preoperatively to postoperatively. In addition, DASH score, Levine score, 2-point discrimination and degree of flexion contracture were assessed.

**Results:**

The patients with A1 annular pulley stenosis showed a significant increase in grip strength 6 months and one year postoperatively. Patients with carpal tunnel syndrome and Dupuytren’s contracture showed no significant difference in grip strength over the course of time. An increase in the percentual grip strength of the thenar in patients with carpal tunnel disease and within the affected finger in A1 annular pulley stenosis was observed over the course of time. The DASH score was significantly lower in all patient cohorts one year postoperatively.

**Conclusion:**

Surgical procedure in carpal tunnel syndrome, A1 annular ligament stenosis and Dupuytren’s contracture improves the functionality of the hand in everyday life. Some areas of the hand seem to compensate other weaker areas in grip strength.

## Introduction

Frequent hand disorders are associated with limitations in grip strength and load distribution. Both factors play an important role in mental health and social interactions [1–4].

Carpal tunnel syndrome is the most common peripheral nerve compression syndrome [5–7]. It impacts the grip strength in a negative way [8]. Other authors observed a drop in grip strength within the first operative month but an improvement after more than 3 months postoperatively. [9] In another study, a 30% higher grip strength was observed 6 months postoperatively [10].

With a lifetime risk of 2–3%, A1 annular pulley stenosis is a common hand pathology [11]. Studies to measure grip strength in patients with A1 annular pulley stenosis with a cylindrical gripping device are still missing. In the literature, a reduced overall grip strength was observed compared to healthy individuals. [11] After surgical treatment, grip strength was seen to be increased 12 weeks postoperatively [12].

Already in 1987, authors stated that the long fingers have the highest proportion of the total strength with a decreasing participation of the fingers from radial to ulnar. The index and middle fingers showed the highest strength values, each with 30 to 33% of the total strength. [2, 13] Only few studies have measured the grip strength of patients pre- and postoperatively and data were above all collected with a Jamar dynamometer [14].

In this study, a cylinder with multiple force sensors and a surrounding sensor mat called manugraphy^®^ (novel GmbH, Munich, Germany) was used. The manugraphy^®^ system with its cylindrical sensor mat detects all vertical forces and is also able to detect load distribution of the hand [15].

In a previous study we calculated a cut-off value of $\ge$ 19 cm between the tip of the middle finger and the proximal palmar crease at the wrist for the use of the larger manugraphy^®^ cylinder as the load distribution depends on the cylinder size [16, 17]. Load distribution differed between the dominant and non-dominant hand in healthy individuals. Additionally, mean and maximum grip strength values were higher for the dominant hand than for the non-dominant hand in right-handed individuals [18].

Aim of this study is the comparison of the grip strength in patients with carpal tunnel syndrome, A1 annular pulley stenosis and Dupuytren’s contracture pre- and postoperatively, in relation to a preexisting control group [18] and versus the healthy contralateral side. Analysis should show if different areas of the hand are especially affected and if operation would lead to a significant increase in hand grip strength and a change in load distribution.

## Materials and methods

### Study design

This study is a monocentric prospective case–control study. The study was conducted according to the guidelines of the Declaration of Helsinki and approved by the Ethics Committee of Friedrich-Alexander-University of Erlangen-Nuremberg, Germany (registration number: 369_15B). Three different groups of patients were enrolled in the Department of Plastic and Hand Surgery, University Hospital Erlangen. The first group included patients with carpal tunnel syndrome, the second one patients with A1 annular pulley stenosis of one finger and the third one patients with Dupuytren’s contracture. Three patients with Dupuytren’s contracture were affected on two rays—on the ring and little finger. All other patients were affected on one ray. Three patients suffered from Dupuytren’s contracture at Tubiana stage 1, three patients stage 2 and one patient stage 3.

To estimate the hypothetical grip strength of each individual's affected hand without the existing pathology, a regression analysis between the grip strength of the dominant and non-dominant hand was performed. For this purpose, a preexisting control group consisting of healthy individuals was analyzed in terms of their grip strength [18]. A linear regression analysis was performed using the values of their dominant and non-dominant hand resulting in a regression formula. This formula could be used for calculating the hypothetical grip strength of the affected hand of the patients without having the hand pathology using the values of the non-affected hand. Informed consent was obtained for every patient included in this study. Each patient received an operation for treating the pathological condition. Data were collected before the operation, 6 weeks, 6 months and one year postoperatively. Every measurement was repeated three times and the mean was documented. The examination was performed in a standardized manner with the patient in an upright sitting position with the shoulder joint in neutral position and the elbow joint flexed 90 degrees [18].

A mean and a maximum value of grip strength was calculated for the different hand areas and for the whole hand out of 3 measure cycles [15, 18, 19]. Exclusion criteria were simultaneous other diseases or previous operations of the hand and bilateral affection. Only adult persons in healthy psychological condition were included.

The DASH score was evaluated in every patient while two-point discrimination and Levine score were documented in all patients with carpal tunnel syndrome. Flexion contracture in all finger joints was measured in the patients with Dupuytren’s contracture.

### Measuring system and selection of the gripping device

The novel’s manugraphy® system (novel biomechanics laboratory, Munich, Germany) is a tool for measurement of the medium and maximum grip force and load distribution of the hand. Measurements are done by two different cylinders**—**one with a diameter of 50 mm and one with a diameter of 65 mm. The length between the fingertip of the middle finger and the distal wrist crease was measured before the first analysis to choose the correct cylinder size. Patients with a length of more than 19 cm used the large cylinder, in case of less the small cylinder was used [18]. The cylinders are wrapped by a sensor mat which detects grip forces between 10 and 600 kPa.

### Surgical procedures

*Carpal tunnel syndrome*: A minimal-invasive mono-portal video-endoscopic carpal tunnel release was carried out under local anesthesia in all patients of this study as standard method in our clinic in primary carpal tunnel syndrome release [20–22]

*A1 annular pulley stenosis*: All patients with A1 annular pulley stenosis received an annular pulley split of the affected ray under local anesthesia.

*Dupuytren’s contracture*: In this study, all patients with Dupuytren’s contracture received a limited aponeurectomy of the affected ray. This involved the open removal of the locally thickened connective tissue strands of the hollow hand.

### Statistical analysis

GraphPad Prism 8.0 (GraphPad Software Inc., La Jolla, CA, USA) was used for statistical analysis. For normal distribution, the Shapiro–Wilk Test was used.

The cross-sectional data of the healthy control group were evaluated with a t-test for connected samples in the case of normal distribution and with a Wilcoxon signed rank sum test in the case of non-normal distribution [18]. The data from the hypothetical/actual grip strength comparison were also evaluated using a t-test.

When evaluating the DASH score and the Levine score, the mean hand strength and percentage strength distribution, the statistical evaluation was carried out using one-way ANOVA for normally distributed data, and the Friedman test was used for data that were not normally distributed.

A score was created for the statistical evaluation of the 2-point discrimination. A 2-point discrimination of > 10 mm was scored with 3 points, a 2-point discrimination of 6 mm-10 mm with 2 points and a 2-point discrimination of < 6 mm with 1 point. Accordingly, the smallest score to be achieved per hand is 10 points, and the largest score to be achieved is 30 points. The statistical evaluation was carried out using the Friedman test and the Wilcoxon signed rank sum test.

In the statistical evaluation of the flexion contracture, the extension deficits of all finger joints of the affected ray were added. For subjects with more than one affected ray, each ray was included in the statistics individually.

Additionally, a post-hoc power analysis with one-way ANOVA (*α* = 0.05) was performed due to the low number of patients in each group. Therefore, G*Power (Heinrich Heine University Düsseldorf, Germany) was used. The statistical power was 15.82% (*β* = 0.84) in the carpal tunnel syndrome group, 42.40% (*β* = 0.58) in the A1 annular pulley stenosis group and 34.80% (*β* = 0.65) in the Dupuytren’s contracture group.

## Results

### Regression analysis

For a regression analysis, data of 40 healthy individuals were additionally included from our previous published study [18]. When determining the regression line, the measured mean total grip strength of the dominant side of the hand was compared with the value of the non-dominant side of the hand. The resulting regression equation is *y* = 0.8506x + 2.3061, where x is the dominant side of the hand and y is the non-dominant side of the hand. The coefficient of determination *R*2 = 0.9015 and the calculated correlation coefficient *R* = 0.949 represent the high linear correlation of the collected data. The determined regression equation was used to assess a possible loss of strength in sick patients and served as the basis for calculating the hypothetical/actual grip strength comparison in the individual patient collectives.

### Carpal tunnel syndrome

In the carpal tunnel syndrome group, a total of 12 subjects were recruited, of which 9 subjects were included (3 drop-outs due to incomplete follow-up). 77.8% were male participants, and the rest was female. The average age was 54.1 years (29–83 years). Five subjects used the small cylinder whereas 4 subjects utilized the large measuring cylinder. Eight patients were right-handed subjects and one patient was left-handed. Four individuals showed the disease on their respective dominant side, and the rest showed the disease on the non-dominant side of the hand.

The patients with carpal tunnel syndrome showed a decrease in the DASH score postoperatively and over time. While a mean value of 31.41 ± 16.4 points could be determined preoperatively, 24.04 ± 18.36 points were counted 6 weeks postoperatively and 12.44 ± 14.74 points 6 months postoperatively. The lowest value was one year postoperatively with a mean of 10.88 ± 14.13 points.

A significant improvement in the DASH score can be observed when comparing the preoperative examination with the examination 6 months (*p* value = 0.0061) as well as one year after the surgery (*p* value = 0.0005). The values 6 weeks after surgery compared to the values one year after surgery also revealed a significant difference with a p value of 0.0370.

### Levine score

Overall, a steady decrease in the symptom severity scale can be seen over all 4 examination times. The highest value with a mean value of 3.10 ± 0.73 could be determined preoperatively. Six weeks (p value = 0.033, mean 1.99 ± 0.81) and 6 months (*p* value = 0.014, mean 1.74 ± 0.81) postoperatively the value is significantly lower compared to preoperatively. Additionally, there is a significant lower value 1 year postoperatively (*p* value = 0.0011, mean 1.49 ± 0.54) compared to preoperatively and a significant difference can be determined between 6 weeks and 1 year postoperatively (*p* value = 0.015).

For the functional status scale, a continuous decrease in mean values can also be shown here. The highest mean value of 2.33 ± 0.80 was obtained at the preoperative examination. Values were significantly lower 1 year postoperatively (*p* value = 0.0117, mean 1.64 ± 0.77). Also, a significantly lower value could be observed after 1 year compared to 6 weeks postoperatively (*p* value = 0.040, mean 2.02 ± 0.85).

### 2-point discrimination

The 2-point discrimination score showed no significant differences between all time points. The preoperative mean score was 12.50 ± 4.72. After 6 weeks postoperatively, the mean value was 11.25 ± 2.76, and after 6 months postoperatively, it was 11.75 ± 4.56. The highest score was determined after one year postoperatively with a mean of 13.25 ± 7.09. A comparison of the 2-point discrimination of the affected side with the unaffected side did not result in any significant statistical differences either. The greatest discrepancy between the affected side and the unaffected side was in favor of the unaffected side at the preoperative time point (affected: 12.50 ± 4.72, unaffected: 10.13 ± 0.35).

**Fig. 1:**
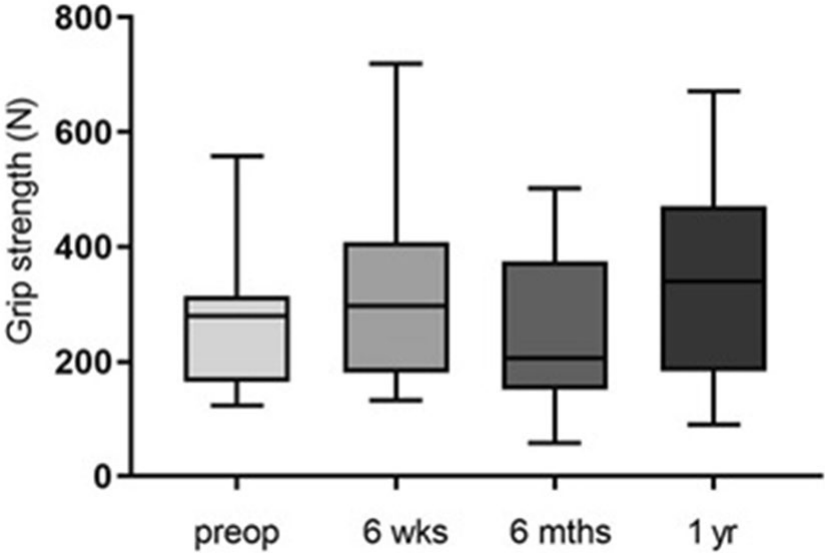
Presentation of the mean grip strength values pre- and postoperatively (*n* = 9). Statistical significance between groups was tested with one-way ANOVA

Figure 1 shows the grip strength at the different time points in carpal tunnel syndrome.

The analysis of the average grip strength of the affected side over all four examination times (Fig. 1) did not reveal any significant differences. The mean value of the preoperative force measurement was 272.0 N ± 129.0 N. The highest force value could be determined 1 year postoperatively (*p* = 0.43) with a mean value of 341.5 N ± 185.0 N.

**Fig. 2:**
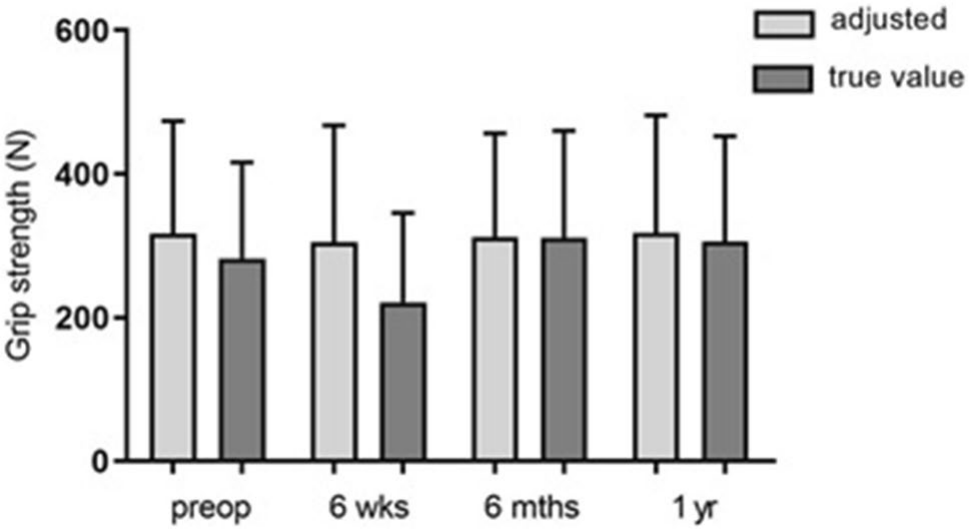
Hypothetical/actual grip strength comparison in the group of right-handers suffering from carpal tunnel syndrome (*n* = 8). Presentation of the respective values at the four different examination times. Statistical significance between groups was tested with paired t-test

The comparison of hypothetical and actual grip strength values (Fig. 2) in the carpal tunnel syndrome group considering the control group revealed a non-significant discrepancy between the hypothetical and actual values at the first two examination times (*p* = 0.08 and *p* = 0.06). Both 6 months postoperatively and 1 year postoperatively, an adjustment of the actual manual strength values to the hypothetical values could be shown.

**Fig. 3:**
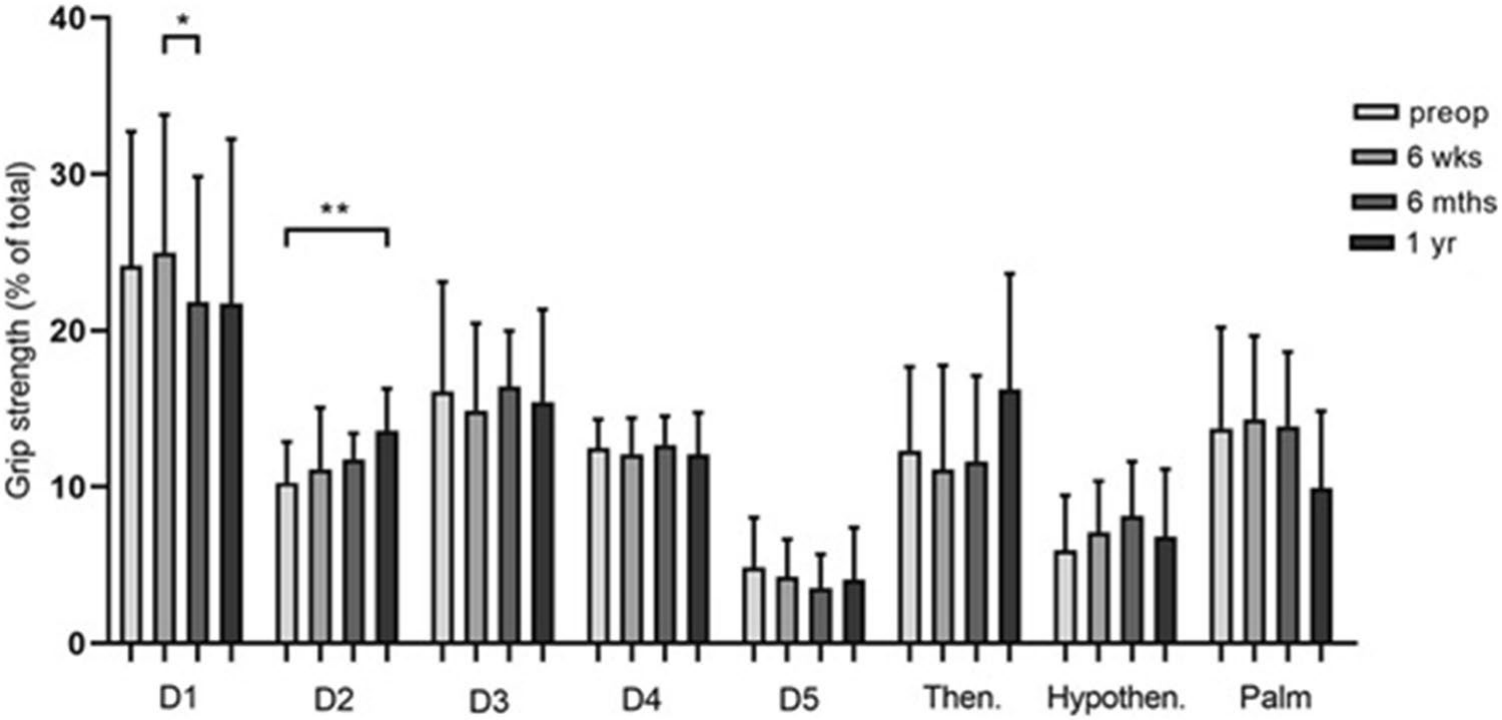
Bar chart showing the percentage grip strength of the respective hand areas D1 = thumb, D2 = index finger, D3 = middle finger, D4 = ring finger, D5 = little finger, Then = thenar, Hypothen = hypothenar pre- and postoperatively (*n* = 9). Statistical significance between groups was tested with one-way ANOVA and Friedman test (* = *p* ≤ 0.05, ** = *p* ≤ 0.01)

When analyzing the load distribution in the different regions of the hand (Fig. 3, Table 1), significant differences in grip strength in individual areas of the hand could be determined. Compared to 6 weeks postoperatively, there is a significant difference 6 months postoperatively (*p* = 0.021). The percentage hand strength of the index finger showed the highest force value 1 year postoperatively (13.60 ± 2.69%). There was a significant difference in percentage of force 1 year postoperatively compared to preoperatively (*p* = 0.005).

**Table 1:** This table shows the percentage grip strength and standard deviation of the different hand areas at different time points in the carpal tunnel syndrome group

Hand area	Preoperatively	6 weeks postoperatively	6 months postoperatively	1 year postoperatively
D1	24.14% ± 8.59%	25.00% ± 8.79%	21.83% ± 8,03%	21.74% ± 10.50%
D2	10.29% ± 2.59%	11.16% ± 3.93%	11.78% ± 1.64%	13.60% ± 2.69%
D3	16.11% ± 7,00%	14.89% ± 5.59%	16.42% ± 3.57%	15.45% ± 5.92%
D4	12.50% ± 1.83%	12.11% ± 2.31%	12.70% ± 1.83%	12.09% ± 2.66%
D5	4.88% ± 3.16%	4.26% ± 2.34%	3.56% ± 2.14%	4.09% ± 3.31%
Thenar	12.35% ± 5.34%	11.14% ± 6.64%	11.66% ± 5.46%	16.24% ± 7.40%
Hypothenar	5.97% ± 3.49%	7.10% ± 3.27%	8.17% ± 3.46%	6.84% ± 4.30%
Palm	13.76% ± 6.46%	14.35% ± 5.32%	13.89% ± 4,76%	9.94% ± 4.90%

### A1 annular pulley stenosis

A total of 15 patients were recruited for the group with A1 annular pulley stenosis, of which a total of 12 patients were included in the study (3 drop-outs due to incomplete follow-up of the patients). 58.3% of the included subjects were male and 41.7% were female. The mean age of the cohort is 61.8 (35–82 years). A total of 5 patients used the small cylinder and accordingly 7 patients used the large cylinder. The average length of the fingertip of middle finger to distal wrist crease was 18.83 cm. All included participants were right-handed. Five of the 12 patients had disease on the non-dominant side and 7 on the dominant side. The location of the affected ray varied. In ten subjects, only one ray was affected, including the thumb (D1) in two subjects, the index finder (D2) in one subject, the middle finger (D3) in four subjects, the ring finger (D4) in 2 subjects, and the small finger (D5) in one subject. Two patients were affected by 2 different rays, one person by D1/D3 and another person by D3/D4.

The evaluation of the DASH questionnaire showed a decrease in the DASH score over all examination times. The highest DASH score of 30.55 ± 17.39 was shown preoperatively. At the second examination point after 6 weeks, the mean value was 19.27 ± 11.66 points. After 6 months, a significant decrease in the DASH score to a mean of 12.21 ± 15.57 was observed (*p* = 0.034) compared to preoperatively. The lowest DASH score could be determined after 1 year postoperatively. Here, the mean was 9.98 ± 14.71. Here, too, there is a significantly lower value compared to the preoperative starting value (*p* = 0.0006).

**Fig. 4:**
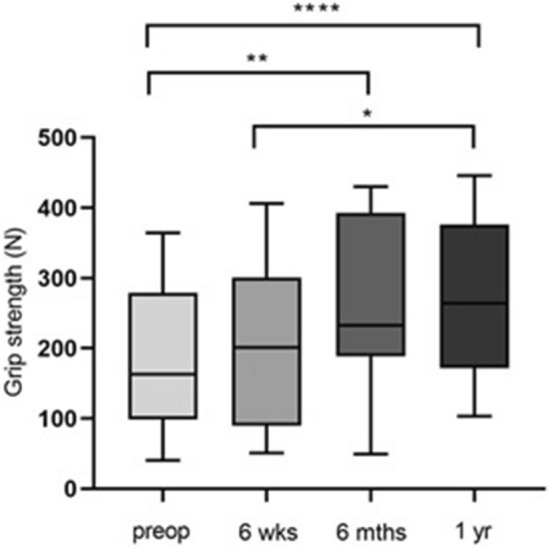
Bar chart showing the mean grip strength values pre- and postoperatively (*n* = 12). Statistical significance between groups was tested with one-way ANOVA (* = *p* ≤ 0.05, ** = *p* ≤ 0.01, **** =  ≤ 0.0001)

Looking at the development of the average hand strength over time (Fig. 4), it can be seen that the highest force value was reached one year postoperatively (272.9 N ± 117.2 N). Significant force differences could be determined on the one hand when comparing the preoperative initial values with the 6 months postoperative measurement (*p* = 0.006) and on the other hand in comparison to the 1-year measurement (*p* ≤ 0.0001). There was also a significant difference in grip strength between the second and fourth time of examination (*p* = 0.024).

**Fig. 5:**
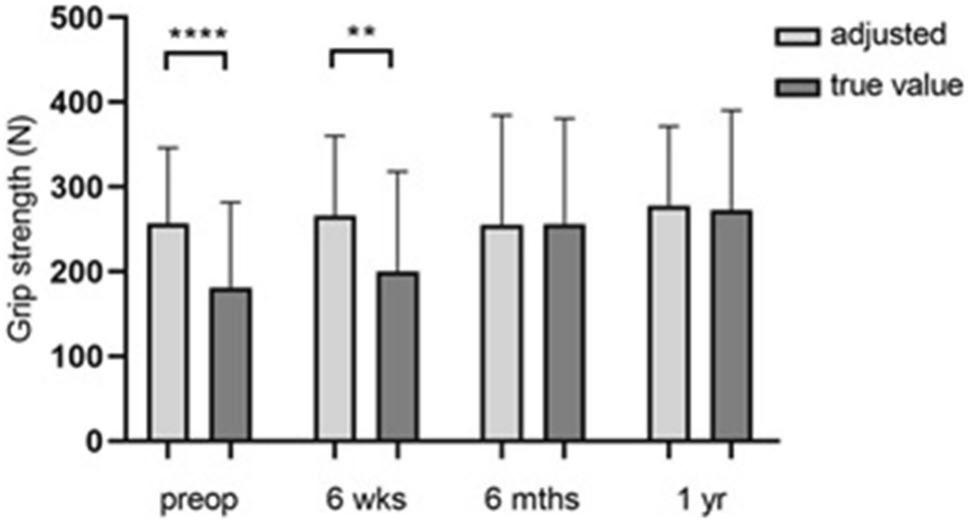
Bar chart showing manual strength (y-axis) for hypothetical and actual values pre- and postoperatively (*n* = 12). Statistical significance between groups was tested with paired t-test (** = *p* ≤ 0.01, **** = *p* ≤ 0.0001)

Figure 5 shows the grip strength for hypothetical and actual values pre- and postoperatively. The preoperative values show the highest discrepancy of all compared values. The calculated hypothetical value was 256.8 N ± 89.23 N and the actual manual strength value was significantly lower with 181.1 N ± 100.8 N. After 6 weeks, there was also a significant difference in force with 266.0 N ± 94.22 N as the hypothetical value and 199.9 N ± 118.1 N as the actual value.

**Fig. 6:**
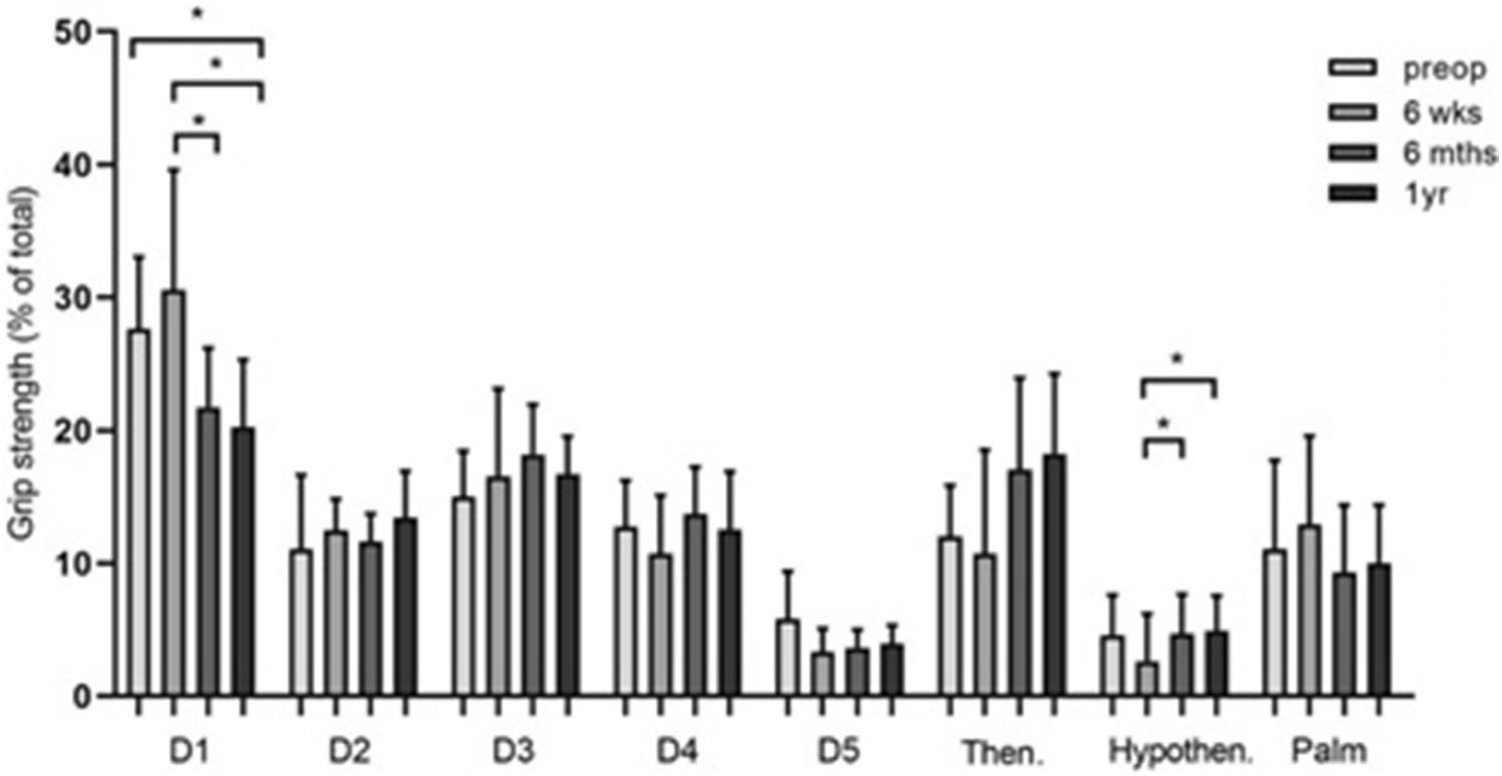
Bar chart showing the percentage hand strength (y-axis) of the respective hand areas (x-axis) D1 = thumb, D2 = index finger, D3 = middle finger, D4 = ring finger, D5 = little finger, Then = thenar, Hypothen = hypothenar pre- and postoperatively (*n* = 6). All enrolled subjects were affected on strand D3. Statistical significance between groups was tested with one-way ANOVA and Friedman test (* = *p* ≤ 0.05)

Figure 6 and Table 2 show the percentage grip strength of the different hand areas at different time points. For statistical reasons, the load distribution for A1 annular pulley stenosis was examined only in the subgroup of patients who were affected on D3 (*n* = 6). Significant grip strength differences over time could be determined for the thumb and the hypothenar. Six weeks postoperatively, a force percentage of 2.64% ± 3.56% could be determined. In the further course, there was a significant increase in the percentage of force to 4.69% ± 2.97% (6 months postoperatively) and 4.90% ± 2.64% (1 year postoperatively) with a respective significance level of *p* = 0.043.

**Table 2:** This table shows the percentage grip strength and standard deviation of the different hand areas at different time points in the A1 annular pulley stenosis group

Hand area	Preoperatively	6 weeks postoperatively	6 months postoperatively	1 year postoperatively	
D1	27.63% ± 5.40%	39.57% ± 9,04%	21.74% ± 4,46%	20.24% ± 5,08%	
D2	11.06% ± 5.59%	12,50% ± 2.32%	11.63% ± 2.08%	13.42% ± 3.51%	
D3	15.03% ± 3.42%	16.57% ± 6.58%	18.20% ± 3.75%	16.72% ± 2.83%	
D4	12.78% ± 3.46%	10.75% ± 4.36%	13.72% ± 3.52%	12.55% ± 4.35%	
D5	5.84% ± 3.56%	3.33% ± 1.75%	3.64% ± 1.35%	3.94% ± 1.39%	
Thenar	12.00% ± 3.88%	10.75% ± 7.78%	17.05% ± 6.89%	18.25% ± 6.02%	
Hypothenar	4.59% ± 3.02%	2.64% ± 3.56%	4.69% ± 2.97%	4.90% ± 2.64%	
Palm	11.08% ± 6.67%	12.90% ± 6.69%	9.32% ± 5.05%	9.97% ± 4.41%	

### Dupuytren’s contracture

The group of patients suffering from Dupuytren's contracture included 10 patients, of which three patients were excluded from the study. All seven included participants were male (100%). The median age is 65.3 years (58–77 years). 71.4% (5 patients) used the large cylinder, and 28.6% (2 people) used the small cylinder. Six patients were right-handed, and one patient was left-handed. For the hypothetical and actual value analysis, the regression equation was used which included solely right-handed individuals. The patients were affected on different hand rays, and some patients were also affected on several rays. Of the participants with only one hand ray affected, one patient was operated on ray D1, one patient on ray D4, and two patients on ray D5. The remaining three patients were affected on both D4 and D5. Two patients were affected on the dominant side of the hand, and five were affected on the non-dominant side of the hand.

Looking at the evaluation of the DASH questionnaire, the preoperative baseline value is 8.84 ± 10.91 points. Six weeks postoperatively, the value was 15.25 ± 17.81 points. In the further course, a lower DASH score can be observed at the examination times 6 months postoperatively and 1 year postoperatively. Six months postoperatively, the determined score is 7.02 ± 5.68 points. At the last examination after 1 year postoperatively, the lowest DASH score with 2.75 ± 3.42 points could be determined. Statistical significance could not be determined between the different examination times.

**Fig. 7:**
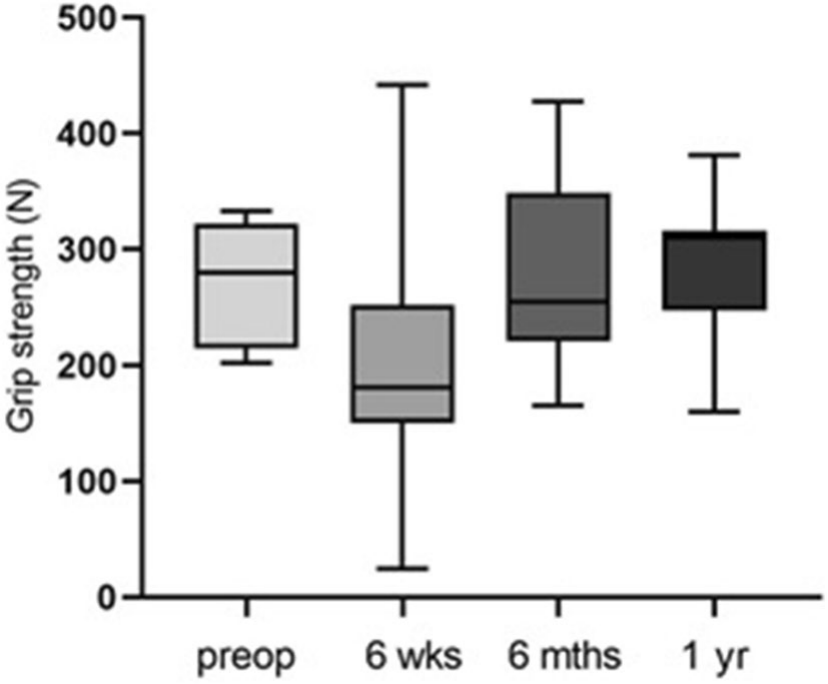
Bar chart showing the mean hand strength values pre- and postoperatively (*n* = 7). Statistical significance between groups was tested with one-way ANOVA

When evaluating the mean hand strength values (Fig. 7), no significance could be determined in a comparison of all examination times.

**Fig. 8:**
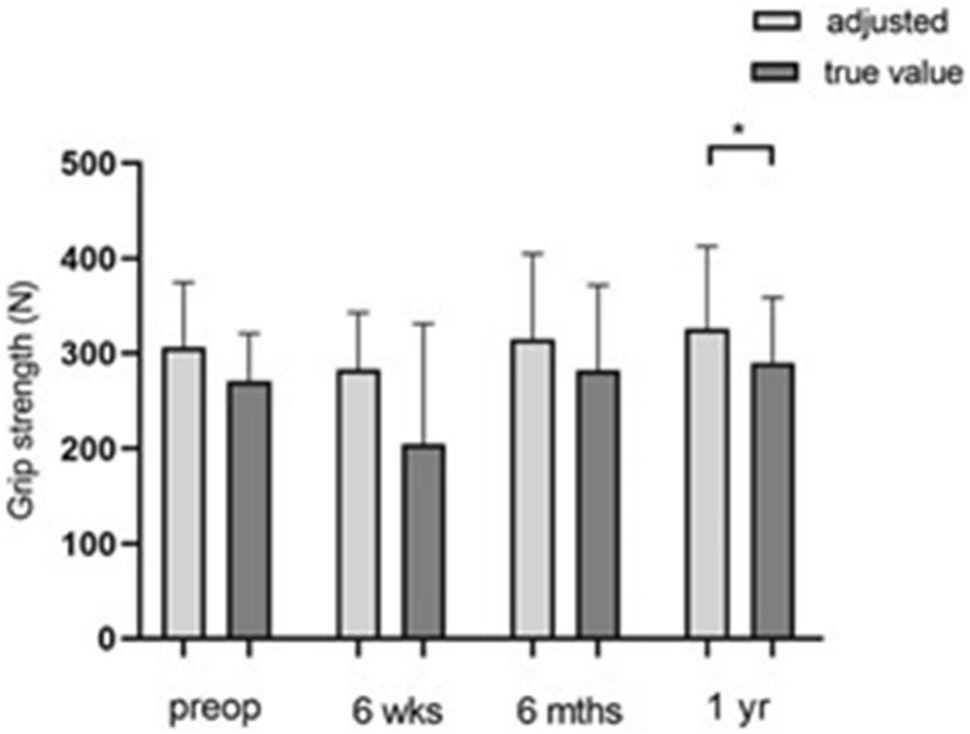
Bar chart showing grip strength for hypothetical and actual values pre- and postoperatively (*n* = 7). Statistical significance between groups was tested with paired t-test (* = *p* ≤ 0.05)

If one compares the actual manual strength values with the calculated hypothetical values (Fig. 8), preoperatively, the hypothetical value is 305.8 N ± 68.45 N, and the actual value is 271.0 N ± 49.54 N. As can be seen from the figure above, the discrepancy in these values tends to increase at the second point in time after 6 weeks postoperatively (p = 0.084). Even at the third examination after 6 months, no significant differences in force could be determined (hypothetical value: 315.0 N ± 89.64 N, actual value: 281.8 N ± 89.79 N, *p* = 0.240). On the other hand, 1 year postoperatively, there were significant force differences when comparing the hypothetical value of 315.4 N ± 87.24 N and the actual value of 289.7 N ± 69.24 N (*p* = 0.0454).

## Discussion

This study shows that the manugraphy^®^ system is well suited for pre- and postoperative control of grip strength and load distribution. It is superior to the widely accepted Jamar dynamometer as it characterizes the grip strength more appropriately due to the measurement of all vertical forces. Another advantage in comparison to the Jamar dynamometer is the possibility to measure different hand areas individually and consequently to identify specific areas of the hand that might compensate other areas of the hand. [15, 18]

The study shows that hand function improved as a result of the operation in all examined diseases in the test period of one year. These results are consistent with the results of other studies that have already been conducted [23, 24]. This study underlines that surgical procedure leads to less symptom burden and an improvement of grip strength in A1 annular pulley stenosis. This is in accordance to findings in the literature. [25] Especially the continuing improvement after 1 year postoperatively confirms the long-term success of the procedure. Half of the included patients were affected at ray D3. As the beam D3 works as a direct antipole to the thumb, this offers an explanation for the fact that the patient group suffered a significant loss of grip strength preoperatively. The still reduced hand strength values 6 weeks postoperatively can certainly also be attributed to the still incomplete tissue healing. The alignment of the hypothetical/actual values after 6 months and 1 year also reflects the above-mentioned increase in strength at these times. It can be assumed that the healing process is complete by this point in time. It appears that a higher proportion of thumb strength is associated with an overall decreased total hand strength. Comparative observations with a cylindrical gripping device are missing so far in the literature.

In carpal tunnel syndrome and Dupuytren’s contracture, results showed no such clear tendencies. This is also consistent with data in the literature where no differences in force pre- and postoperatively were found [14, 26]. No improvement in two-point discrimination after surgical treatment of carpal tunnel syndrome could be demonstrated while some authors found a significant improvement in two-point discrimination up to 24 months after surgical intervention. Others described a high variability of results, which is influenced by the individual perception ability of the patients [27–29]. The results of this study regarding the mean grip strength did not reveal any significant differences between the individual examination times. In studies conducted so far, there are conflicting opinions as to whether there is a loss of grip strength in patients with carpal tunnel syndrome and how an operation affects the development of grip strength. Some authors could show a reduction in grip strength with carpal tunnel syndrome while others could not observe any decrease in grip strength compared to a healthy control group. [30–33] A possible reason could be that carpal tunnel syndrome leads to a continuous motor damage and furthermore to a reduced grip strength.

New modalities of perfusion measurements such as hyperspectral imaging, thermography or Indocyanin green near infrared angiography might be a valuable extension to manugraphy to further delineate the potential effects of microcirculation after operative inventions as has been shown for other hand and plastic surgical entities [34–41].

Regarding Dupuytren’s contracture, a possible reason for the little effect on hand strength could be the fact that MCP, PIP and DIP joints are usually flexed during gripping. [26]

In our previous study, we were able to show on the basis of healthy subjects that the ring finger and little finger represent the fingers with the lowest percentage of total strength. [18] In our group of patients, there was only one patient who was not affected on the ring or little finger. Therefore, the small influence on the total force may be due to this.

## Limitations

The significance of the data collected in this study is limited by the small number of participants due to the specified inclusion criteria. Furthermore, the healthy subjects were much younger, and the proportion of women in the healthy group of subjects was significantly higher as in the trial groups. Additionally, only a few patients were affected on the same ray. Power analysis showed that higher numbers of patients would be necessary for satisfactory conclusions. Yet, due to the mentioned strict exclusion criteria, the number of patients is unfortunately limited in this monocentral study. Nevertheless, we think that the findings of this study are important and should be taken into account as there is no comparable study in the literature to date, especially regarding the A1 annular pulley stenosis group.

In the future, a longer follow-up would be of interest for obtaining even more precise data regarding long-term course of grip strength and load distribution postoperatively.

## Conclusion

In summary, hand function has improved as a result of the respective operation. This study shows that certain areas of the hand are compensated in terms of force by other areas of the hand. Manugraphy^®^ as a measuring instrument has proven to be a reliable system for force measurement, even in the postoperative process.

## References

[1] Kim JK, Park MG, Shin SJ (2014). What is the minimum clinically important difference in grip strength?. Clin Orthop Relat Res.

[2] Radhakrishnan S, Nagaravindra M (1993). Analysis of hand forces in health and disease during maximum isometric grasping of cylinders. Med Biol Eng Comput.

[3] Talsania JS, Kozin SH (1998). Normal digital contribution to grip strength assessed by a computerized digital dynamometer. J Hand Surg Br.

[4] Taekema DG (2010). Handgrip strength as a predictor of functional, psychological and social health A prospective population-based study among the oldest old. Age Ageing.

[5] Padua L (2016). Carpal tunnel syndrome: clinical features, diagnosis, and management. Lancet Neurol.

[6] Unglaub F (2010). Bilateral atypical muscles causing acute bilateral carpal tunnel syndrome in recreational climber. Arch Orthop Trauma Surg.

[7] El-Karabaty H (2005). The effect of carpal tunnel release on median nerve flattening and nerve conduction. Electromyogr Clin Neurophysiol.

[8] Baker NA (2013). Effect of carpal tunnel syndrome on grip and pinch strength compared with sex- and age-matched normative data. Arthritis Care Res (Hoboken).

[9] Young VL (1992). Grip strength before and after carpal tunnel decompression. South Med J.

[10] Fernandes CH (2013). Carpal tunnel syndrome with thenar atrophy: evaluation of the pinch and grip strength in patients undergoing surgical treatment. Hand (N Y).

[11] Langer D (2017). Evaluating hand function in clients with trigger finger. Occup Ther Int.

[12] Dierks U, Hoffmann R, Meek MF (2008). Open versus percutaneous release of the A1-pulley for stenosing tendovaginitis: a prospective randomized trial. Tech Hand Up Extrem Surg.

[13] Amis AA (1987). Variation of finger forces in maximal isometric grasp tests on a range of cylinder diameters. J Biomed Eng.

[14] Zyluk A, Jagielski W (2007). The effect of the severity of the Dupuytren's contracture on the function of the hand before and after surgery. J Hand Surg Eur.

[15] Muhldorfer-Fodor M (2014). Grip force monitoring on the hand: Manugraphy system versus Jamar dynamometer. Arch Orthop Trauma Surg.

[16] Mühldorfer-Fodor SZ, Harms C, Cristalli A, Kalpen A, Kundt G, Mittlmeier T, Prommersberger KJ (2012). Pressure distribution within the hand during cylinder grip. J Hand Surg.

[17] Lee JW, Rim K (1991). Measurement of finger joint angles and maximum finger forces during cylinder grip activity. J Biomed Eng.

[18] Cai A (2018). Force distribution of a cylindrical grip differs between dominant and nondominant hand in healthy subjects. Arch Orthop Trauma Surg.

[19] Garkisch A (2022). Influence of the flexor digitorum superficialis tendon transfer on grip strength. J Hand Surg Eur.

[20] Eisenhardt SU (2010). Retrospective analysis of 242 patients whose carpal tunnels were released using a one-port endoscopic procedure: superior results of early intervention. J Plast Surg Hand Surg.

[21] Hessenauer MET, Horch RE (2019). Endoscopic carpal tunnel release via a monoportal approach. Zentralbl Chir.

[22] Polykandriotis E, Premm W, Horch RE (2007). Carpal tunnel syndrome in young adults–an ultrasonographic and neurophysiological study. Minim Invasive Neurosurg.

[23] Barros MFFH (2015). Evaluation of surgical treatment of carpal tunnel syndrome using local anesthesia. Revista brasileira de ortopedia.

[24] Mallick A, Clarke M, Kershaw CJ (2007). Comparing the outcome of a carpal tunnel decompression at 2 weeks and 6 months. J Hand Surg Am.

[25] Uzel K (2022). Isolated A1 pulley release surgery for trigger finger leads to significant increase in tip-to-tip pinch strength. J Orthop Sci.

[26] Garkisch A (2020). Dynamic manugraphy as a promising tool to assess the outcome of limited aponeurectomy in patients with Dupuytren’s contracture. Front Med (Lausanne).

[27] Shurr DG, Blair WF, Bassett G (1986). Electromyographic changes after carpal tunnel release. J Hand Surg Am.

[28] Kanatani T (2014). Electrophysiological assessment of carpal tunnel syndrome in elderly patients: one-year follow-up study. J Hand Surg Am.

[29] Lundborg G, Rosén B (2004). The two-point discrimination test – time for a re-appraisal?. J Hand Surg (Edinburgh, Scotland).

[30] Atalay N (2011). The impact of disease severity in carpal tunnel syndrome on grip strength, pinch strength, fine motor skill and depression. J Phys Ther Sci.

[31] Fernández-de-Las-Peñas C (2009). Bilateral deficits in fine motor control and pinch grip force in patients with unilateral carpal tunnel syndrome. Exp Brain Res.

[32] Singh GK, Srivastava S (2020). Grip strength of occupational workers in relation to carpal tunnel syndrome and individual factors. Int J Occup Saf Ergon.

[33] Afifi M, Santello M, Johnston JA (2012). Effects of carpal tunnel syndrome on adaptation of multi-digit forces to object texture. Clin Neurophysiol.

[34] Aslan-Horch EC (2022). Effects of different pressure levels in topical negative pressure application-analysis of perfusion parameters in a clinical skin model using multimodal imaging techniques. J Clin Med.

[35] Frank K (2022). Improving the safety of DIEP flap transplantation: detailed perforator anatomy study using preoperative CTA. J Pers Med..

[36] Geierlehner A (2022). Intraoperative blood flow analysis of DIEP vs ms-TRAM flap breast reconstruction combining transit-time flowmetry and microvascular indocyanine green angiography. J Pers Med.

[37] Promny, D., et al., *[Paradoxical Perfusion Reaction of the contralateral Hand after Hand MRI].* Handchir Mikrochir Plast Chir, 2022. 10.1055/a-1738-9906 35158399

[38] Müller-Seubert W (2021). Novel imaging methods reveal positive impact of topical negative pressure application on tissue perfusion in an in vivo skin model. Int Wound J.

[39] Müller-Seubert W (2022). A personalized approach to treat advanced stage severely contracted joints in Dupuytren's disease with a unique skeletal distraction device-utilizing modern imaging tools to enhance safety for the patient. J Pers Med.

[40] Müller-Seubert W (2022). Evaluation of the influence of short tourniquet ischemia on tissue oxygen saturation and skin temperature using two portable imaging modalities. J Clin Med.

[41] Müller-Seubert W (2022). Intra- and early postoperative evaluation of malperfused areas in an irradiated random pattern skin flap model using indocyanine green angiography and near-infrared reflectance-based imaging and infrared thermography. J Pers Med.

